# Clinical and Radiological Characterization of Central Nervous System Involvement in Nocardiosis: A 20-Year Experience

**DOI:** 10.7759/cureus.52950

**Published:** 2024-01-25

**Authors:** Razvan M Chirila, Dana Harris, Vivek Gupta, Donna J Hata, Claudiu Matei, Salvador Alvarez, Adrian G Dumitrascu

**Affiliations:** 1 Internal Medicine, Mayo Clinic, Jacksonville, USA; 2 Radiology, Mayo Clinic, Jacksonville, USA; 3 Microbiology, Mayo Clinic, Jacksonville, USA; 4 Neurological Surgery, Lucian Blaga University, Sibiu, ROU; 5 Hospital Medicine, Mayo Clinic, Jacksonville, USA

**Keywords:** neurological signs and symptoms, nocardia species, antibiotic susceptibility, bacterial cns infection, cerebral nocardiosis

## Abstract

Background

This study aimed to present the clinical and radiological characteristics and the outcomes of patients with *Nocardia *infection of the central nervous system (CNS).

Methodology

We conducted a retrospective review of patients aged 18 and older admitted between August 1998 and November 2018 with culture-proven nocardiosis and CNS involvement.

Results

Out of 110 patients with nocardiosis, 14 (12.7%) patients had CNS involvement. The median age was 54.5 (27, 86) years, and 12 (85.7%) patients were male. Overall, 12 (85.7%) patients were immunosuppressed on high doses of glucocorticoids; seven (50%) patients were solid organ transplant recipients. Only eight (57.1%) patients had neurological symptoms at presentation, and the rest were diagnosed with CNS involvement after imaging surveillance. Three distinct radiologic patterns were identified, namely, single or multiple abscesses, focal cerebritis, and small, septic embolic infarcts. All isolates of *Nocardia *were susceptible to trimethoprim/sulfamethoxazole and amikacin, with susceptibility to linezolid and carbapenems being 90.9% and 79.5%, respectively. Despite receiving antibiotic therapy, six (42.8%) patients died, most of them within weeks of initial admission. All surviving patients underwent prolonged antimicrobial therapy until the resolution of MRI abnormalities. All solid organ transplant recipients recovered.

Conclusions

*Nocardia* CNS infection was a rare condition, even among a large, immunosuppressed patient population. CNS imaging surveillance is paramount for immunosuppressed patients with nocardiosis, as CNS involvement influences the choice and duration of therapy. *Nocardia* antibiotic susceptibility varied widely between strains and the empiric therapy should consist of multiple classes of antimicrobials with CNS penetration. Mortality was high, but all solid organ transplant recipients recovered.

## Introduction

*Nocardia *species are Gram-positive, partially acid-fast, catalase-positive bacteria that are found in water, soil, and other organic matter and grow as filamentous, branching rods [1,2]. Inhalation of *Nocardia* is the main route of transmission, although it may penetrate damaged skin. Disseminated infection preferentially affects the lungs, soft tissue, and central nervous system, and bacteremia is rare [3,4]. T lymphocytes have the primordial role in the protective immune response against this organism, but deficits in B-cell function have been implicated in alterations of the cell-mediated immune response against *Nocardia* [5]. As such, this infection is commonly seen in immunosuppressed individuals such as transplant recipients, patients on chronic high-dose glucocorticoid therapy, patients with lymphoma, patients with human immunodeficiency virus (HIV)/acquired immunodeficiency syndrome (AIDS), and patients with defects in the humoral and innate immune systems. [5] These patients comprise more than 60% of all patients with nocardiosis [6].

*Nocardia *species have a known propensity toward invasion and reproduction within the central nervous system (CNS). CNS involvement frequency is variable with up to 33% of patients in some series [1]. Animal experiments have shown that some *Nocardia* strains have surface receptors that recognize and bind sites on capillary endothelial cells in the CNS. The progression and clearance of the CNS infection depends on the virulence of the strain as well as the inoculum of *Nocardia species* infecting the brain [6-9]. Previous brain pathology is also considered a risk factor for CNS nocardiosis [1,9].

CNS infection may be present with headache, seizure, focal neurological deficits, and non-specific complaints, or it can be insidious, showing no initial neurological signs or symptoms [1,5]. CNS nocardiosis should always be ruled out in patients with disseminated or pulmonary disease, especially if patients are immunosuppressed [1]. Brain magnetic resonance imaging (MRI) or contrast-enhanced computed tomography (CT) is recommended in such cases [1]. CNS nocardiosis is a rare condition associated with a remarkably high one-year mortality rate (20-40%) [10]. The majority of the available literature regarding this condition is based on case reports and small case series. Recently, a systematic review of all case reports and series was published [10]. We present our 20-year experience regarding the diagnosis and management of patients with this rare condition.

## Materials and methods

Study design and participants

We designed a single-center, retrospective cohort study evaluating patients from a tertiary academic center, Mayo Clinic in Jacksonville, Florida (MCF). The study was approved by the Mayo Clinic Institutional Review Board (ID: 17-010028). We described the study methods as well as the clinical characteristics and outcomes of all patients with nocardiosis diagnosed at our center in a previous paper [11]. The present study provides a detailed analysis of patients with CNS nocardiosis.

We identified all microbiology specimens positive for *Nocardia *species that were collected from patients evaluated at our institution between August 1998 and November 2018. A total of 202 individual patients had one or more culture-positive specimens and their medical records were reviewed. The definition of invasive disease required a positive culture for *Nocardia* species and the presence of clinical signs of infection and/or radiological evidence of organ involvement. Of the 202 patients, 110 met the criteria for invasive disease. The remaining 92 patients were considered to be colonized with *Nocardia*. Colonization [1] was defined as a patient having a positive culture in a specimen obtained from a non-sterile site in the absence of clinical or radiological evidence of infection. Colonized patients were excluded from the analysis. We reviewed additional clinical, microbiological, radiological, and therapeutical features of patients with invasive disease. After a thorough medical record review, we identified patients with a possible CNS infection with *Nocardia*. For this study, two groups of patients were defined as having CNS *Nocardia* infection. The first group was patients with a culture-positive specimen collected from a brain abscess, meningeal biopsy, or cerebrospinal fluid (CSF). The second group of patients had *Nocardia* species isolated from a specimen other than CNS but had typical CNS nocardiosis radiological findings. The patients in the second group were started on specific anti-*Nocardia *therapy and were followed for resolution of the clinical and radiological findings.

Demographic and clinical data

Patient data were extracted from the MCF electronic medical record, initially from Cerner (Cerner Corporation North Kansas City, MO) and after October 16, 2018, from Epic (Epic Systems Corporation Verona, WI). The data were entered and stored in a clinical database provided by RedCap (Vanderbilt University, Nashville, TN).

Demographic information, comorbidities, immune status, bacteriological information (*Nocardia *species, antibiotic susceptibility), site of infection, clinical symptoms, chest and CNS imaging characteristics, and treatment outcomes were recorded for these patients. An independent neuroradiologist reinterpreted all brain MRIs of the patients with CNS *Nocardia* involvement and described the different radiological patterns.

Specific information was recorded for transplanted patients (type of transplant, antirejection medications used). Patient expiration date and date of the last clinic follow-up were recorded and used to calculate the survival outcomes.

Microbiology

After reviewing morphology on Gram stain and modified acid-fast staining, the MCF microbiology laboratory identified the organisms as “possible *Nocardia* species.” Further speciation, initially using 16S rRNA sequencing and then matrix-assisted laser desorption/ionization time-of-flight mass spectrometry analysis, and subsequent antibiotic sensitivity testing using broth microdilution were finalized at Mayo Clinic Reference Laboratory in Rochester, MN.

## Results

A total of 110 patients with invasive nocardiosis were identified, and 74 (67.3%) patients had brain imaging performed within one month of the diagnosis. Overall, 36 (32.7%) cases of invasive nocardiosis had no brain imaging in our system due to various reasons: two lung transplant patients expired before CNS imaging was performed, 17 chronic lung disease patients were treated successfully by pulmonologists, 11 patients had limited skin and soft tissue infections that recovered with therapy, and five cases were followed elsewhere after diagnosis. In three cases that recovered with therapy, brain imaging was recommended but not performed. Out of 74 patients who underwent brain imaging, 60 (81%) were initially deemed immunosuppressed (47 (63.5%) were solid organ transplant recipients, and 13 (17.5%) had either autoimmune or neoplastic disorders on therapy).

Clinical characteristics

In total, 14 patients with CNS *Nocardia *involvement were identified. The median age was 54.5 (27, 86) years, and 12 (85.7%) patients were males. Overall, 12 (85.7%) patients were immunosuppressed; all 12 were on chronic glucocorticoid therapy either as part of their antirejection regimen (n = 8), immunomodulators for systemic lupus erythematosus (n = 2), or had a prior diagnosis of brain tumor treated with dexamethasone (n = 2). One patient had stem cell transplantation, and seven (50%) patients were solid organ transplant recipients (five kidney transplants, one combined kidney-pancreas transplant, and one lung transplant). The time from solid organ transplant to *Nocardia *diagnosis varied from 50 to 2,353 days with a median (interquartile range) of 484 (283-1,311) days. All solid organ transplant patients were on antirejection medications with tacrolimus and/or mycophenolic acid derivatives. Only two of seven solid organ transplant patients were taking trimethoprim-sulfamethoxazole prophylaxis at diagnosis.

Neurological symptoms on presentation were found in only eight (57.1%) patients, including altered mentation (n = 6, 42%), gait disturbance (n = 2, 14.2%), headache (n = 2, 14.2%), and speech disturbance (n = 4, 28.5%). All six (42%) non-transplant patients had neurological symptoms. Only one of the seven solid organ transplant recipients had neurological symptoms, and the other six were diagnosed with CNS involvement after surveillance imaging despite having no neurological symptoms. Four (28.5%) patients had fever at presentation, and four (28.5%) patients had respiratory symptoms such as cough, dyspnea, and hemoptysis. A total of 12 (85.7%) patients were also diagnosed with pulmonary nocardiosis. Two patients had *Nocardia *endocarditis and *Nocardia *bacteremia. For case descriptions and clinical characteristics, see Table 1.

**Table 1:** Case descriptions and characteristics of patients with central nervous system nocardiosis. CNS imaging pattern by MRI: A, abscess; B, ventriculitis; C, septic emboli. BAL = bronchoalveolar lavage; CNS = central nervous system; CT = computed tomography; F = female; M = male; MRI = magnetic resonance imaging

Case	Age at diagnosis(years)/Sex	Clinical presentation	Comorbid conditions	Case description	MRI CNS imaging pattern	Involved organs	Fluid/Tissue specimen	*Nocardia *species	Total therapy duration	Outcome
1	59/M	Submandibular gland infection	Kidney transplant	Cultures from salivary gland biopsy grew *Nocardia*. Brain and lung masses were found. A lung mass biopsy culture also grew *Nocardia*. The patient was treated with antibiotics until the resolution of the brain MRI findings	A	Lung, salivary gland, brain	Submandibular gland biopsy	N. cyriacigeorgica	16 months	Recovered
2	69/M	Cough, skin nodules	Lung transplant, chronic kidney disease, diabetes mellitus	Right upper lobe cavitary lesion yielded *Nocardia *on BAL culture. Bone and brain imaging abnormalities and skin nodules were present. The patient was treated with antibiotics until the resolution of the brain MRI findings	B	Lung, skin, bone,	BAL	N. pseudobrasileinsis	18 months	Recovered
3	27/M	Fever, chills, post-renal transplant lymphocele	Kidney transplant	The patient was treated for infected peritransplant lymphocele. Blood cultures grew *Nocardia*; tricuspid vegetation was found; brain MRI had septic emboli; brain biopsy cultures had no growth. The patient was treated until the resolution of the brain MRI findings	C	Blood, brain	Blood	N. farcinica	24 months	Recovered
4	48/M	Fever, encephalopathy, skin nodules	Glioblastoma	A patient with glioblastoma post-chemotherapy. The patient had new cavitary lung infiltrates; a skin lesion grew *Nocardia* on biopsy; brain MRI showed new multiple lesions. The patient was treated with intravenous antibiotics, but due to a poor prognosis associated with his brain tumor, he elected hospice care and later expired under hospice care	B	Lung, brain, blood, skin	Skin lesion biopsy	N. brasiliensis	NA	Expired after 15 days
5	66/M	Fever, Fatigue, Lethargy	Systemic Lupus Erythematosus	The patient was transferred from an outside facility for encephalopathy, brain, and lung masses. Brain biopsy grew *Nocardia*. The patient expired despite therapy	A	Lung, brain	Brain lesion biopsy	N. asteroides	NA	Expired after 21 days
6	42/M	Headaches, slurred speech	Kidney transplant, diabetes mellitus	The patient was diagnosed with pulmonary nocardiosis with BAL cultures. The patient was non-adherent to therapy and was readmitted with neurological symptoms. The brain abscess was aspirated and grew *Nocardia*. Due to further non-adherence, his lesion progressed; the patient needed craniotomy with abscess debridement. The patient was treated until the resolution of the brain MRI findings	A	Lung, brain	Brain lesion biopsy	N. beijingensis	18 months	Recovered
7	52/F	Fever, encephalopathy	Hodgkin’s disease, bone marrow transplant, graft versus host disease	The patient had new pulmonary infiltrates and a new brain abscess on brain MRI. BAL culture grew *Nocardia*. The patient expired despite therapy	A	Lung, brain, blood	Blood	N. cyriacigeorgica	NA	Expired after 90 days
8	44/M	Headache, gait disturbance	Alcohol use disorder	Brain MRI was positive for cerebellar abscess. Abscess biopsy culture grew *Nocardia*	A	Lung, brain	Brain lesion biopsy	N. abscessus	12 months	Recovered
9	82/M	Encephalopathy, generalized weakness	Bioprosthetic aortic valve	A patient with a bioprosthetic valve for aortic stenosis and recent hemorrhagic strokes was found to have prosthetic valve endocarditis with *Nocardia *bacteremia, septic brain emboli, and hemorrhagic transformation of embolic strokes. The patient expired despite therapy.	C	Bioprosthetic valve, brain, blood	Blood	N. farcinica	NA	Expired after 21 days
10	86/M	Expressive aphasia, headaches, hemoptysis, skin nodules	Meningioma status post-radiotherapy	A patient with non-operative enlarging meningioma causing brain compression treated with radiation and glucocorticoids. He had new brain abscesses on repeat brain MRI, new cavitary lung nodules, and multiple retroperitoneal, mesenteric, and abdominal nodules. Cultures of an abdominal wall mass grew *Nocardia*. The patient expired despite therapy	A	Lung, brain, skin, mesentery	Skin lesion biopsy	N. farcinica	NA	Expired after 90 days
11	57/M	Generalized weakness, cough	Kidney transplant, HIV on therapy	The patient had a right lung mass; *Nocardia *grew on BAL culture. Contrast-enhanced CT had numerous abscesses. The patient was treated until the resolution of brain CT findings	B	Lung, brain	BAL	N. cyriacigeorgica	12 months	Recovered
12	79/M	Fatigue, Dyspnea	Kidney Transplant	The patient had multiple pulmonary nodules and BAL culture yielded *Nocardia*. Brain lesions were found on MRI. The patient was treated until the resolution of the brain MRI findings.	A	Lung, brain	BAL	N. abscessus	12 months	Recovered
14	50/M	Fever, cough, dyspnea	Kidney and pancreas Transplant	Pulmonary infiltrates were found and *Nocardia *was isolated from sputum cultures. Brain MRI showed multiple abscesses. The patient was treated until the resolution of the brain MRI findings	A	Lung, brain	Sputum	N. cyriacigeorgica	12 months	Recovered
14	29/F	Encephalopathy, gait disturbance	Systemic lupus erythematosus	Contrast-enhanced brain CT showed multiple abscesses; chest CT showed pulmonary infiltrates. *Nocardia *was isolated from CSF culture. The patient expired despite therapy	A	Lung, brain	CSF	N. farcinica	NA	Expired after 30 days

Microbiological characteristics

Four (28.5%) patients had positive cultures on specimens obtained from CNS: three patients had brain lesion biopsy, and one had *Nocardia* isolated from CSF. Another patient had a negative brain biopsy culture. Ten (71.4%) patients had a positive culture specimen isolated from a site other than CNS, as well as concomitant radiological and clinical evidence of CNS nocardiosis.

There were four (28.5%) isolates of *Nocardia cyriacigeorgica*, four (28.5%) of *Nocardia farcinica*, and one isolate each of *Nocardia pseudobrasiliensis*, *Nocardia brasiliensis*, *Nocardia asteroides*,* Nocardia abscessus*, and *Nocardia beijingensis*. The initial specimen source and *Nocardia *species with corresponding antibiotic susceptibility are listed in Table 2. The antibiotic susceptibility profile for *Nocardia *species is listed in Table 3.

**Table 2:** Nocardia species and susceptibility to antibiotics. S = susceptible; I = intermediate; R = resistant; NT = not tested; BAL = bronchoalveolar lavage; CSF = cerebrospinal fluid; TMP/SMX = trimethoprim/sulfamethoxazole

Case	Specimen source	*Nocardia* organism	TMP/SMX	Ceftriaxone	Amoxicillin/Clavulanic acid	Imipenem	Amikacin	Tobramycin	Ciprofloxacin	Moxifloxacin	Clarithromycin	Minocycline	Linezolid	Cefepime	Doxycycline
1	Salivary gland	Nocardia cyriacigeorgica	S	S	I	S	S	S	R	R	R	I	S	S	I
2	BAL	Nocardia pseudobrasiliensis	S	R	I	R	S	S	S	S	S	R	I	R	R
3	Blood	Nocardia farcinica	S	R	S	S	NT	R	S	NT	R	S	NT	R	NT
4	Skin	Nocardia brasiliensis	S	R	S	S	S	S	R	NT	R	I	S	R	I
5	Brain	Nocardia asteroides	S	S	NT	R	S	S	R	NT	S	R	NT	NT	NT
6	Brain	Nocardia asteroides	S	S	NT	S	S	S	NT	NT	S	S	S	NT	NT
7	Blood	Nocardia cyriacigeorgica	S	I	R	S	S	S	R	NT	R	I	S	R	I
8	Brain	Nocardia abscessus	S	S	R	S	S	S	R	R	R	S	S	S	S
9	Blood	Nocardia farcinica	S	R	S	S	S	R	R	S	R	I	S	R	I
10	Skin	Nocardia farcinica	S	R	S	S	S	R	R	NT	R	I	S	R	I
11	BAL	Nocardia cyriacigeorgica	S	S	I	S	S	S	I	S	R	I	S	R	I
12	BAL	Nocardia abscessus	S	S	S	I	S	S	R	I	R	S	S	S	S
13	Sputum	Nocardia cyriacigeorgica	S	I	R	S	S	S	R	R	R	I	S	R	I
14	CSF	Nocardia farcinica	S	I	NT	S	S	R	S	NT	R	I	NT	NT	NT

**Table 3:** Antibiotic susceptibility profile of Nocardia species.

Tested medication	Tested isolates (n)	Susceptible (%)	Intermediate (%)	Resistant (%)
Trimethoprim/Sulfamethoxazole	14	100%	0%	0%
Amikacin	13	100%	0%	0%
Linezolid	11	90.9%	9.0%	0%
Imipenem	14	78.5%	7.1%	14.2%
Tobramycin	14	71.4%	0%	28.5%
Amoxicillin/Clavulanic acid	11	45.4%	27.2%	27.2%
Ceftriaxone	14	42.8%	21.4%	35.7%
Moxifloxacin	7	42.8%	14.2%	42.8%
Minocycline	14	28.5%	57.1%	14.2%
Cefepime	11	27.2%	0%	72.7%
Ciprofloxacin	13	23.0%	7.6%	69.2%
Clarithromycin	14	21.4%	0%	78.5%
Doxycycline	10	20%	70%	10%

Imaging characteristics

MRI of the brain was done in 12 (85.7%) patients and intravenous gadolinium contrast was administered in eight patients. Two patients were evaluated with contrast-enhanced CT scan only. Radiological findings are listed in Table 4.

**Table 4:** Radiological findings on brain imaging.

Characteristic	Patients, N (%)
Single lesion	5 (35.7%)
Multiple lesions	9 (64.2%)
Infratentorial-only lesions	1 (7.1%)
Supratentorial-only lesions	5 (35.7%)
Supratentorial and infratentorial lesions	8 (57.1%)
Lesion >5 mm	9 (64.2%)
Lesions <5 mm	5 (35.7%)

**Figure 1:**
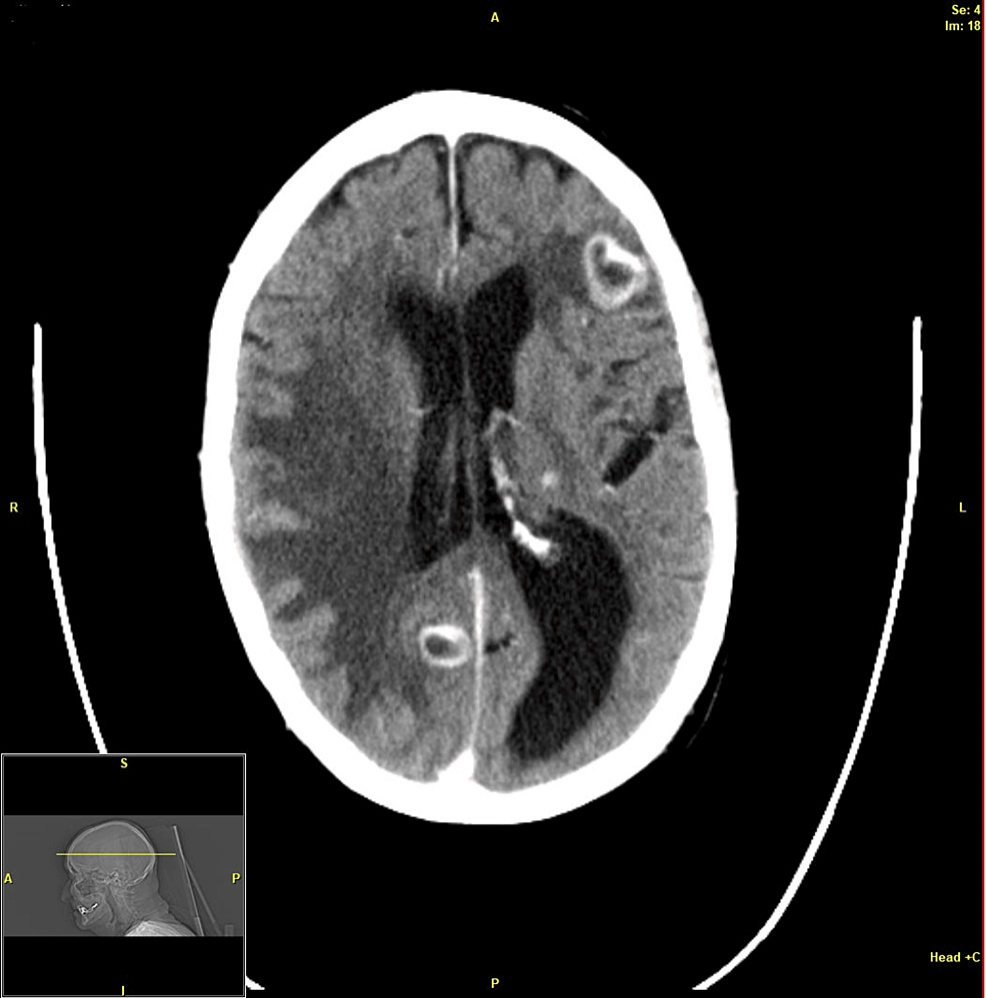
CT of the head with contrast enhancement showing ring-enhancing subcortical lesions.

On an unenhanced CT scan, brain lesions were either absent or appeared as focal or ill-defined subcortical hypodensities, with a typical ring enhancement of the abscess after iodinated contrast was given (Figure 1). The hypodensities either resolved or became less conspicuous upon antibiotic treatment and did not calcify upon healing.

On MRI, CNS lesions included abscesses (round/oval lesions larger than 5 mm) with typical ring enhancement and vasogenic edema or smaller lesions with variable enhancement and minimal edema. Local magnetic susceptibility was nearly always seen either on the initial or on follow-up MRI in the brain lesions of nocardiosis indicating microscopic hemorrhage at some stage in their evolution. The MR findings in our series could be classified into three distinct patterns.

Pattern A: Single or Multiple Abscesses

**Figure 2:**
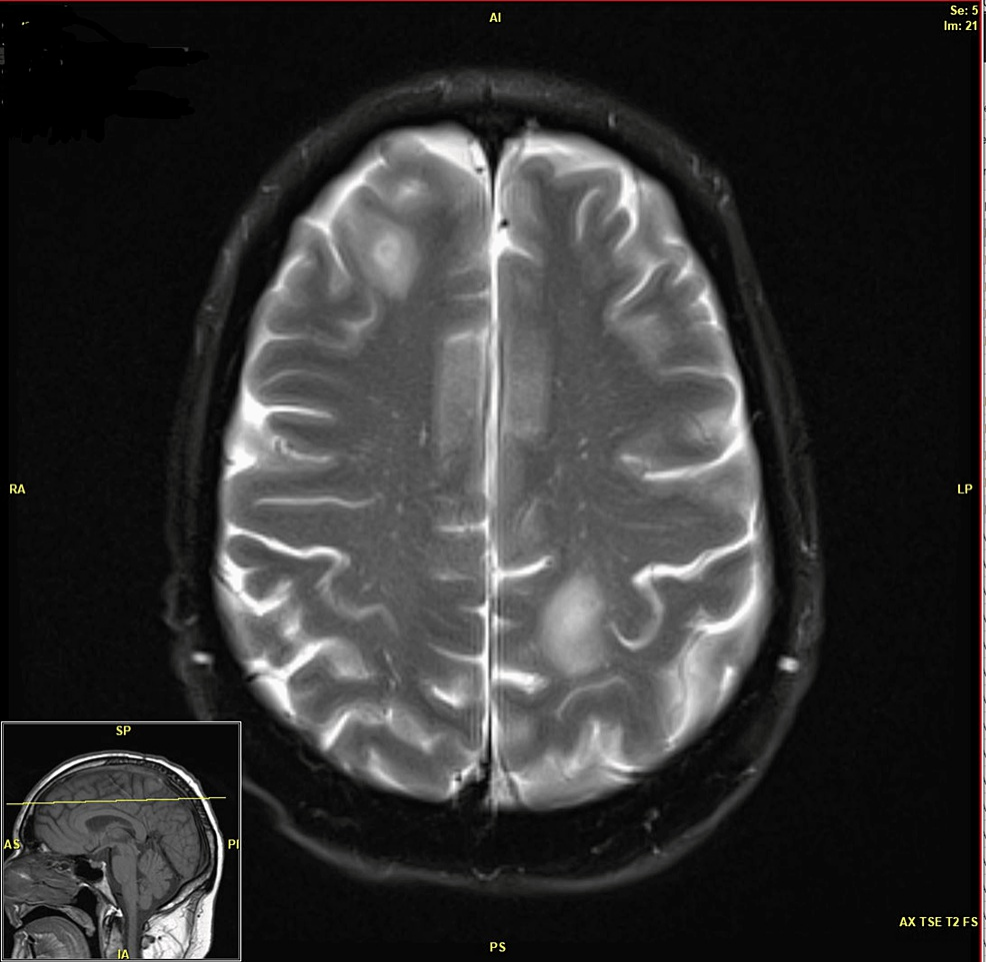
MRI pattern A.

This was the most frequently (nine patients, 64.2%) encountered form of brain involvement. The abscesses were almost always subcortical in location and showed typical peripheral ring enhancement and central high T2 signal and restricted diffusion. The cerebellar location of the abscess was seen in two patients. Most of these abscesses were round or oval, measuring from 5 mm to over 3 cm, with an occasional abscess appearing lobulated or dumbbell-shaped. The surrounding vasogenic edema was variable but almost always present when the lesions were larger than 10 mm (Figure 2).

Pattern B: Focal Cerebritis

**Figure 3:**
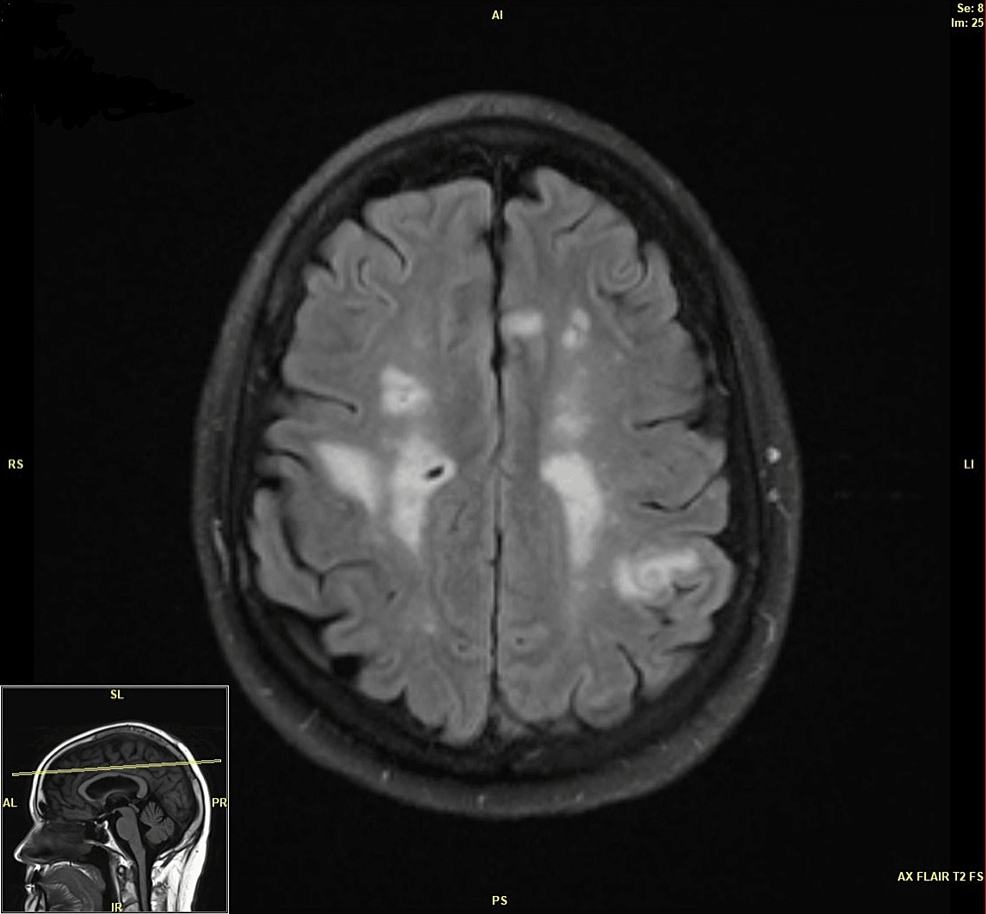
MRI pattern B.

This pattern was present in three (21.4%) patients. MRI displayed single or multiple, 5 mm or less, centrally T2 dark lesions with variable enhancement leading to either ring or target appearance, usually with minimal surrounding vasogenic edema (Figure 3).

Pattern C: Small Septic Embolic Infarcts

**Figure 4:**
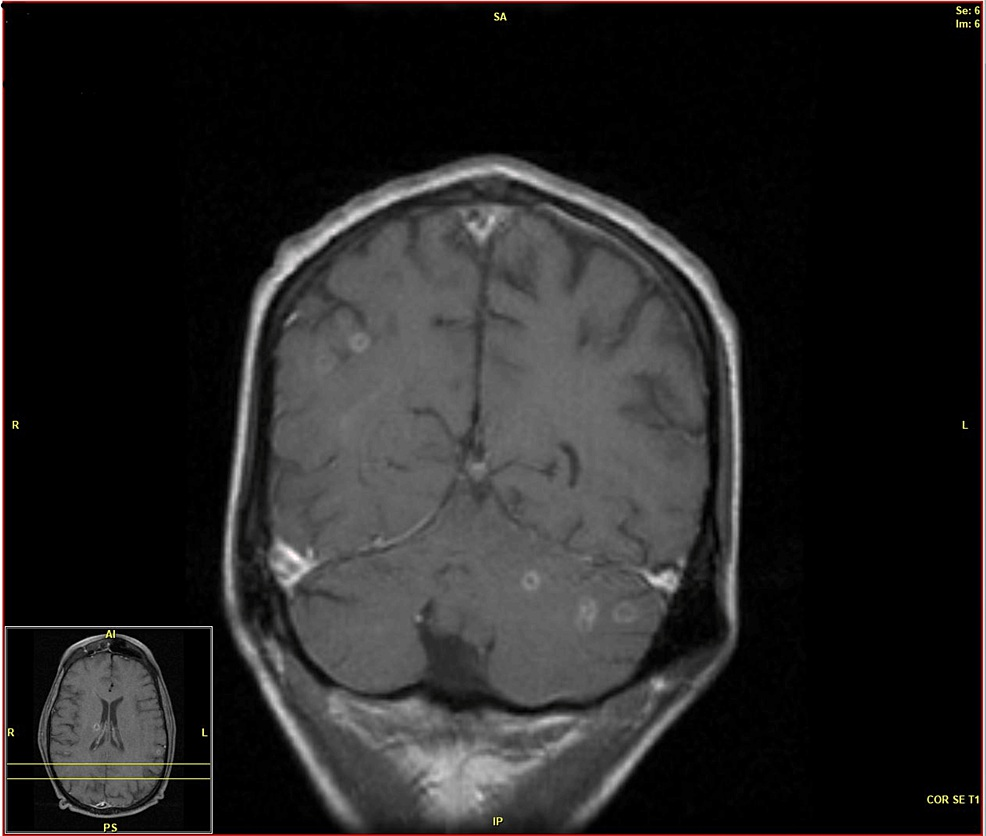
MRI pattern C.

This pattern was present in two (14.2%) patients. Scattered cortical and subcortical punctate (3-5 mm) lesions of restricted diffusion were seen in two patients. This pattern was seen in patients with severe pulmonary nocardiosis and disseminated infection (in both cases, endocarditis was present). Ventriculitis was present in one patient, and, in the other patient, there were diffusely scattered punctate (<3 mm) lesions on diffusion-weighted imaging throughout the brain in a “miliary” pattern. Many of these lesions also showed enhancement possibly due to the development of early cerebritis (Figure 4).

Treatment and outcomes

All patients were initially treated in the hospital with a combination of parenteral antibiotics. Most patients were started on broad-spectrum empirical antimicrobials (based on their original presentation) until Nocardia species were isolated in cultures. Therapy was then changed to empiric anti-nocardial antibiotics with two or three drugs with CNS penetration. Typically, this involved intravenous trimethoprim/sulfamethoxazole associated with a second drug such as a carbapenem, linezolid, ceftriaxone and in some cases a third drug such as a fluoroquinolone or a tetracycline. All 14 patients received inpatient treatment with trimethoprim/sulfamethoxazole, nine of the patients received a carbapenem, four received ceftriaxone, two received linezolid, two received doxycycline, and one received ciprofloxacin. One patient required desensitization to trimethoprim/sulfamethoxazole. One patient developed bone marrow toxicity from linezolid, following which the drug was discontinued. Amikacin was not used in our patient population.

The final antibiotic choice included trimethoprim/sulfamethoxazole together with one or two additional antibiotics. There was a high variability in susceptibility to the second or third antibiotic and, after the antibiogram resulted, therapeutic regimens were tailored accordingly, with most patients receiving a carbapenem as a second drug.

Six (42.8%) patients expired. One patient expired at 15 days (a patient with glioblastoma), three patients expired at 30 days (two patients with systemic lupus and one with Nocardia bioprosthetic aortic valve endocarditis), and two patients expired at 90 days (one patient with recurrent Hodgkin’s disease and one with unresectable meningioma). Eight (57.1%) patients recovered with appropriate therapy. The patient with alcohol use disorder and cerebellar abscess recovered after 12 months of therapy. All seven solid organ transplant recipients also recovered. MRI findings resolved after 12 months in three patients, and three others required a longer duration of therapy for resolution (16 to 24 months). Another patient underwent craniotomy with surgical debridement with extension of therapy to a total of 18 months.

## Discussion

A total of 110 patients were identified at our institution as having *Nocardia *infection, out of more than 200,000 admissions from August 1998 to November 2018. We identified 14 cases with CNS *Nocardia* involvement that comprised 12.7% of all cases of patients with nocardiosis and 18.9% of all patients with nocardiosis who underwent brain imaging during that time frame. CNS *Nocardia *infection had a low incidence at our institution despite MCF providing care for numerous patients with solid organ and stem cell transplants, malignancy, and autoimmune disorders.

Overall, 85% of patients with CNS nocardiosis were immunosuppressed, with all of them receiving glucocorticoids at diagnosis. Literature data suggests that glucocorticosteroid therapy increases the risk of *Nocardia *infection in both solid organ transplant and non-transplant patients [3,11-14]. In a review study of 206 cases of CNS nocardiosis done by Meena et al., glucocorticosteroid therapy was the most important predisposing factor for CNS infection, with 56% of patients receiving this medication [10]. Half of all patients in our series were solid organ transplant recipients on antirejection medication, and an additional 14% of patients had autoimmune disorders requiring high doses of immunosuppression, another recognized risk factor. In the above-mentioned review study, 26.2% of patients had a solid organ transplant, with a total of 44% of patients being immunosuppressed on chemotherapy or antirejection medication [10]. An additional risk factor is previous brain pathology [1,9], and 14% of our patients also had this condition in their history.

A significant proportion (42.9%) of patients in our study, including the majority of transplant patients, did not have neurological complaints at presentation. This initial absence of clinical neurological findings despite positive CNS imaging was described previously. In the European review that included 30 patients with CNS nocardiosis, Coussement et al. also noted that 13 (43.3%) patients had no neurological signs or symptoms at diagnosis [3]. Our finding emphasizes the expert opinion recommendation, i.e., brain imaging should be done in every immunocompromised patient suspected or diagnosed with nocardiosis [11]. The discovery of CNS involvement would have major clinical and survival implications as it influences the choice and duration of antibiotic therapy [11]. Promptly started, broad-spectrum, effective, and prolonged antibiotic therapy is needed to treat this condition which is associated with a very high mortality rate. Once CNS lesions are found, serial brain imaging should be used to assess their response to therapy. Based on serial MRI follow-ups, half of the patients in our series had their therapy prolonged beyond 12 months until the resolution of imaging findings [11].

The extent of brain involvement was monitored with MRI and in two patients with contrast enhanced CT due to the presence of MRI contraindication. Single or multiple abscesses (pattern A) were the most common finding at 64.2%. Meena et al. noted brain abscesses in 86.9% of the reviewed patients [10]. Focal cerebritis (pattern B) probably represents an earlier stage before the development of abscesses, as typically seen in many other abscess-forming CNS infections. Small embolic infarcts were seen as a complication of *Nocardia *endocarditis.

Our laboratory identified the *Nocardia *species based on 16S rRNA sequencing. The most common species were *Nocardia cyriacigeorgica* and *Nocardia farcinica* at 28% each. *Nocardia farcinica* was also the most prevalent species (39.6%) in the review by Meena et al. [10] and in a* Nocardia* brain abscess series (37.5%) published by Campioli et al. [15]. Previous studies in a mouse model indicated that *Nocardia farcinica* is more virulent than other strains and leads to disseminated disease and CNS nocardiosis more frequently [3,16-19]. *Nocardia farcinica* has a resistance pattern that can make treatment difficult, characteristically being resistant to third-generation cephalosporins [1,2,20]. In our study, all *Nocardia farcinica* strains were resistant to ceftriaxone.

Antimicrobial susceptibilities varied widely between strains, as listed in Table 3. Empirical therapy consisted of multiple classes of antimicrobials with CNS activity once *Nocardia *species were identified in cultures. This therapy was readily converted to targeted therapy based on the susceptibility profiles. Most strains were susceptible to trimethoprim-sulfamethoxazole, amikacin, and carbapenems, comparable to other studies [13,21]. In our study, *Nocardia *species sensitivity for trimethoprim-sulfamethoxazole, amikacin, and imipenem was 100%, 100%, and 78.5%, respectively, and Meena et al. found an 88.9%, 82.8, and 80.6% corresponding sensitivity [10]. Similar to their finding of only 58.7% sensitivity to ceftriaxone, we also encountered a 48.2% sensitivity to this antibiotic. Despite having a larger patient sample in their study, the sensibility pattern seems to be similar. We found that amikacin was not utilized in our cohort, possibly due to concern about its nephrotoxic effect. The literature suggests that trimethoprim-sulfamethoxazole is the cornerstone of treatment for *Nocardia *infections, also being the drug of choice for cerebral nocardiosis due to its good CNS penetration [11,22-24]. All strains isolated in our study were susceptible to trimethoprim-sulfamethoxazole, and all patients received this drug. The available literature recommends treatment for at least 12 months in patients with CNS involvement to prevent relapse [4,11,15,24,25]. Antibiotic therapy was administered for at least 12 months in patients who recovered from the infection, with three (21.4%) patients requiring a longer duration of therapy, as mentioned above. Two of our patients with solid organ transplants were on trimethoprim-sulfamethoxazole for prophylaxis and still had breakthrough *Nocardia *infection with a strain that proved to still be sensitive and to respond to the therapeutic dose of trimethoprim-sulfamethoxazole. This interesting feature was previously described [12].

Mortality in our retrospective cohort was high at 42.8%, but still in line with what was previously reported for this condition. In an expert opinion article, Lebeaux et al. noted a reported mortality range between 20% and 40% [11]. Patients who did not survive either presented late to our institution or had a guarded prognosis related to their underlying condition (relapsed Hodgkin’s disease, glioblastoma multiforme, advanced systemic lupus erythematosus, inoperable brain tumor, multiple hemorrhagic strokes from endocarditis). They expired within weeks of presentation despite the initiation of anti-nocardial antibiotic therapy.

Two retrospective studies suggested that surgical debridement associated with antimicrobial therapy is superior to either approach alone concerning mortality rate [9,10]. However, the diagnostic or therapeutic neurosurgical approach is controversial in the absence of randomized studies. Expert opinion recommendations favor an individualized approach that would balance patient risks, abscess size and location, and the surgical procedure risk [10]. Only four (28.5%) patients in our study had to undergo brain abscess diagnostic biopsy. A single patient, having progression of his brain abscess due to non-compliance with antibiotic therapy, had to undergo abscess evacuation. The rest of our study patients had either no indication (multiple, small brain lesions) or were not clinically stable for neurosurgical intervention.

All our seven (50%) patients with solid organ transplants and CNS nocardiosis recovered with the appropriate therapy. In our institution, these patients are followed closely and get expedited access to care if they develop new symptoms. They were admitted and underwent prompt diagnostic testing with isolation of *Nocardia *species from cultures. They were cared for by a transplant team and a dedicated transplant infectious disease specialist both inpatient and outpatient. This aggressive treatment approach may explain the unexpected positive outcomes in this otherwise fragile patient population.

Older studies listed advanced HIV disease as an important risk factor for nocardiosis [8]. However, the reported prevalence of HIV infection was low in recent studies (Meena et al. listing eight out of 206 patients, 3.8%) [10], most likely due to the use of highly active antiretroviral therapy in the last two decades [2,26]. In our study we only had one HIV-positive patient (7.1%); he was also a kidney transplant recipient with an undetectable HIV viral load.

Our study has several limitations. It was conducted in a single center, and it is a retrospective observational cohort study with no controls for comparison. Our institution is a tertiary center, a reference cancer therapy center, and a transplant center that cares for sicker patients than most facilities; this vulnerable patient population is closely monitored and benefits from an expedited evaluation pathway. These factors may limit the generalization of our findings.

## Conclusions

In our study, *Nocardia *CNS infection was found to be a rare condition, even among a large, immunosuppressed patient population.

As previously recommended by expert opinion, CNS imaging surveillance is very important in immunosuppressed patients with systemic *Nocardia* infection, as results will influence the choice and therapy duration. Serial imaging is needed to assess the response to antibiotic therapy. Three distinct radiologic patterns can be identified by CNS imaging: cerebral abscess, focal cerebritis, and septic embolic infarcts. Although having distinct features, the septic emboli and cerebritis patterns may represent different phases of CNS infection in the progression toward the final stage of multiple cerebral abscesses.

*Nocardia *antibiotic susceptibility varies widely between strains, and empirical therapy should consist of multiple classes of antimicrobials with CNS activity. This broad-spectrum therapy should be readily converted to a targeted therapy based on the susceptibility profiles (most strains were sensitive to trimethoprim-sulfamethoxazole, amikacin, and carbapenems).

## References

[1] Chen SC, Watts MR, Maddocks S, Sorrell TC (2020). Nocardia species. Mandell, Douglas, and Bennett's Principles and Practice of Infectious Diseases 9th Edition.

[2] Conville PS, Witebsky FG (2015). Nocardia, Rhodococcus, Gordonia, Actinomadura, Streptomyces, and other aerobic Actinomycetes. Manual of Clinical Microbiology.

[3] Coussement J, Lebeaux D, van Delden C (2016). Nocardia infection in solid organ transplant recipients: a multicenter European case-control study. Clin Infect Dis.

[4] Wilson JW (2012). Nocardiosis: updates and clinical overview. Mayo Clin Proc.

[5] Restrepo A, Clark NM (2019). Nocardia infections in solid organ transplantation: guidelines from the Infectious Diseases Community of Practice of the American Society of Transplantation. Clin Transplant.

[6] Beaman BL, Beaman L (1994). Nocardia species: host-parasite relationships. Clin Microbiol Rev.

[7] Beaman BL, Beaman L, Kjelstrom JA, Ogata SA (1994). Bacteria and neurodegeneration. Neurodegenerative Diseases.

[8] Beaman BL, Boiron P, Beaman L, Brownell GH, Schaal K, Gombert ME (1992). Nocardia and nocardiosis. J Med Vet Mycol.

[9] Anagnostou T, Arvanitis M, Kourkoumpetis TK, Desalermos A, Carneiro HA, Mylonakis E (2014). Nocardiosis of the central nervous system: experience from a general hospital and review of 84 cases from the literature. Medicine (Baltimore).

[10] Meena DS, Kumar D, Bohra GK, Midha N, Garg MK (2022). Clinical characteristics and treatment outcome of central nervous system nocardiosis: a systematic review of reported cases. Med Princ Pract.

[11] Harris DM, Dumitrascu AG, Chirila RM (2021). Invasive nocardiosis in transplant and nontransplant patients: 20-Year experience in a tertiary care center. Mayo Clin Proc Innov Qual Outcomes.

[12] Lebeaux D, Coussement J, Bodilsen J, Tattevin P (2021). Management dilemmas in Nocardia brain infection. Curr Opin Infect Dis.

[13] Steinbrink J, Leavens J, Kauffman CA, Miceli MH (2018). Manifestations and outcomes of nocardia infections: comparison of immunocompromised and nonimmunocompromised adult patients. Medicine (Baltimore).

[14] Peleg AY, Husain S, Qureshi ZA (2007). Risk factors, clinical characteristics, and outcome of Nocardia infection in organ transplant recipients: a matched case-control study. Clin Infect Dis.

[15] Corsini Campioli C, Castillo Almeida NE, O'Horo JC, Challener D, Go JR, DeSimone DC, Sohail MR (2021). Clinical presentation, management, and outcomes of patients with brain abscess due to Nocardia species. Open Forum Infect Dis.

[16] Wang HL, Seo YH, LaSala PR, Tarrand JJ, Han XY (2014). Nocardiosis in 132 patients with cancer: microbiological and clinical analyses. Am J Clin Pathol.

[17] Valdezate S, Garrido N, Carrasco G, Medina-Pascual MJ, Villalón P, Navarro AM, Saéz-Nieto JA (2017). Epidemiology and susceptibility to antimicrobial agents of the main Nocardia species in Spain. J Antimicrob Chemother.

[18] Desmond EP, Flores M (1993). Mouse pathogenicity studies of Nocardia asteroides complex species and clinical correlation with human isolates. FEMS Microbiol Lett.

[19] Kumar VA, Augustine D, Panikar D, Nandakumar A, Dinesh KR, Karim S, Philip R (2014). Nocardia farcinica brain abscess: epidemiology, pathophysiology, and literature review. Surg Infect (Larchmt).

[20] Schlaberg R, Fisher MA, Hanson KE (2014). Susceptibility profiles of Nocardia isolates based on current taxonomy. Antimicrob Agents Chemother.

[21] Hamdi AM, Fida M, Deml SM, Abu Saleh OM, Wengenack NL (2020). Retrospective analysis of antimicrobial susceptibility profiles of Nocardia species from a tertiary hospital and reference laboratory, 2011 to 2017. Antimicrob Agents Chemother.

[22] Brown-Elliott BA, Biehle J, Conville PS (2012). Sulfonamide resistance in isolates of Nocardia spp. from a US multicenter survey. J Clin Microbiol.

[23] Smego RA Jr, Moeller MB, Gallis HA (1983). Trimethoprim-sulfamethoxazole therapy for Nocardia infections. Arch Intern Med.

[24] Wallace RJ Jr, Septimus EJ, Williams TW Jr, Conklin RH, Satterwhite TK, Bushby MB, Hollowell DC (1982). Use of trimethoprim-sulfamethoxazole for treatment of infections due to Nocardia. Rev Infect Dis.

[25] Lerner PI (1996). Nocardiosis. Clin Infect Dis.

[26] Minero MV, Marín M, Cercenado E, Rabadán PM, Bouza E, Muñoz P (2009). Nocardiosis at the turn of the century. Medicine (Baltimore).

